# Required properties for markers used to calculate unbiased estimates of the genetic correlation between populations

**DOI:** 10.1186/s12711-018-0434-6

**Published:** 2018-12-14

**Authors:** Yvonne C. J. Wientjes, Mario P. L. Calus, Pascal Duenk, Piter Bijma

**Affiliations:** Animal Breeding and Genomics, Wageningen University and Research, 6700 AH Wageningen, The Netherlands

## Abstract

**Background:**

Generally, populations differ in terms of environmental and genetic factors, which can create differences in allele substitution effects between populations. Therefore, a single genotype may have different additive genetic values in different populations. The correlation between the two additive genetic values of a single genotype in two populations is known as the additive genetic correlation between populations and thus, can differ from 1. Our objective was to investigate whether differences in linkage disequilibrium (LD) and allele frequencies of markers and causal loci between populations affect the bias of the estimated genetic correlation. We simulated two populations that were separated by 50 generations and differed in LD pattern between markers and causal loci, as measured by the LD-statistic *r*. We used a high marker density to represent a high consistency of LD between populations, and lower marker densities to represent situations with a lower consistency of LD between populations. Markers and causal loci were selected to have either similar or different allele frequencies in the two populations.

**Results:**

Our results show that genetic correlations were underestimated only slightly when the difference in allele frequencies between the two populations was similar for the markers and the causal loci. A lower marker density, representing a lower consistency of LD between populations, had only a minor effect on the underestimation of the genetic correlation. When the difference in allele frequencies between the two populations was not similar for markers and causal loci, genetic correlations were severely underestimated. This bias occurred because the markers did not predict accurately the relationships at causal loci.

**Conclusions:**

For an unbiased estimation of the genetic correlation between populations, the markers should accurately predict the relationships at the causal loci. To achieve this, it is essential that the difference in allele frequencies between populations is similar for markers and causal loci. Our results show that differences in LD phase between causal loci and markers across populations have little effect on the estimated genetic correlation.

**Electronic supplementary material:**

The online version of this article (10.1186/s12711-018-0434-6) contains supplementary material, which is available to authorized users.

## Background

Alleles in different populations are often expressed in different environments and genetic backgrounds. Because of genotype by environment interactions and non-additive genetic effects, these differences result in different allele substitution effects between populations [1–3]. In addition, the set of loci that underlie a trait can differ between populations. Therefore, a single genotype may have different additive genetic values in different populations [2, 4]. For each population, the additive genetic value is the product of the genotype, which is measured as the allele count at each locus, multiplied by the allele substitution effects for that population. The additive genetic correlation between two populations is the correlation between the two additive genetic values of a single genotype in the two populations and may differ considerably from 1.

Knowledge of the genetic correlation between populations helps to understand the differences and similarities in genetic architecture of complex traits between populations [5, 6]. For both genomic prediction and genome-wide association studies, combining information from populations is an attractive approach to increase the accuracy of estimated genetic values or the power to identify quantitative trait loci. This is especially the case when the number of individuals with marker genotypes and phenotypes in a population is limited. For both genomic prediction and genome-wide association studies, the genetic correlation between populations determines the added benefit of combining information from multiple populations [5, 7, 8]. Therefore, the genetic correlation between populations is an important parameter in human studies e.g. [5, 9], as well as in animal and plant breeding e.g. [10, 11].

To estimate a genetic correlation between two populations, it is essential to know the relationships between individuals from the two populations. Traditionally, relationships between individuals are based on pedigree information, which generally is only available within a population. The current availability of genome-wide marker panels has opened up new opportunities to estimate genetic correlations between populations of distantly related individuals, such as between breeds e.g. [10, 12], lines [13], sub-populations e.g. [11], or ethnicities e.g. [5, 9]. Genetic correlations between populations can be estimated using methods based on genomic relationships [10], random regression on marker genotypes [14, 15], or summary statistics of genome-wide association studies [6, 16]. Wientjes et al. [17] showed that it is possible to obtain an unbiased estimate of the genetic correlation from genomic relationships based on causal loci.

Because causal loci are generally unknown, genomic relationships have to be based on marker information. It is expected that this creates bias in the estimates of genetic correlation, because the strength and phase of linkage disequilibrium (LD) between causal loci and markers differ between populations in humans [18], livestock [19, 20], and plants [21, 22]. Due to imperfect LD between causal loci and markers, not all the genetic variance is explained by the markers, which can further distort the estimation of genetic correlations [16, 23]. In contrast to the expectations, a simulation study with populations that had different LD patterns showed that the estimated genetic correlation between populations based on marker information was only slightly biased [7]. This contrast indicates that the impact of differences in LD patterns between populations on the estimated genetic correlation remains unclear.

The objective of this study was to investigate whether differences in LD patterns between populations and differences in allele frequencies of markers and/or causal loci between populations affect bias of the estimated genetic correlation. We simulated two populations that were separated by 50 generations using scenarios that differed in consistency of LD between populations, defined as the correlation in LD phase between the two populations, and in allele frequencies of markers and causal loci between the populations. We used marker-based relationship matrices to estimate the genetic correlation.

## Methods

### Population structure

**Fig. 1:**
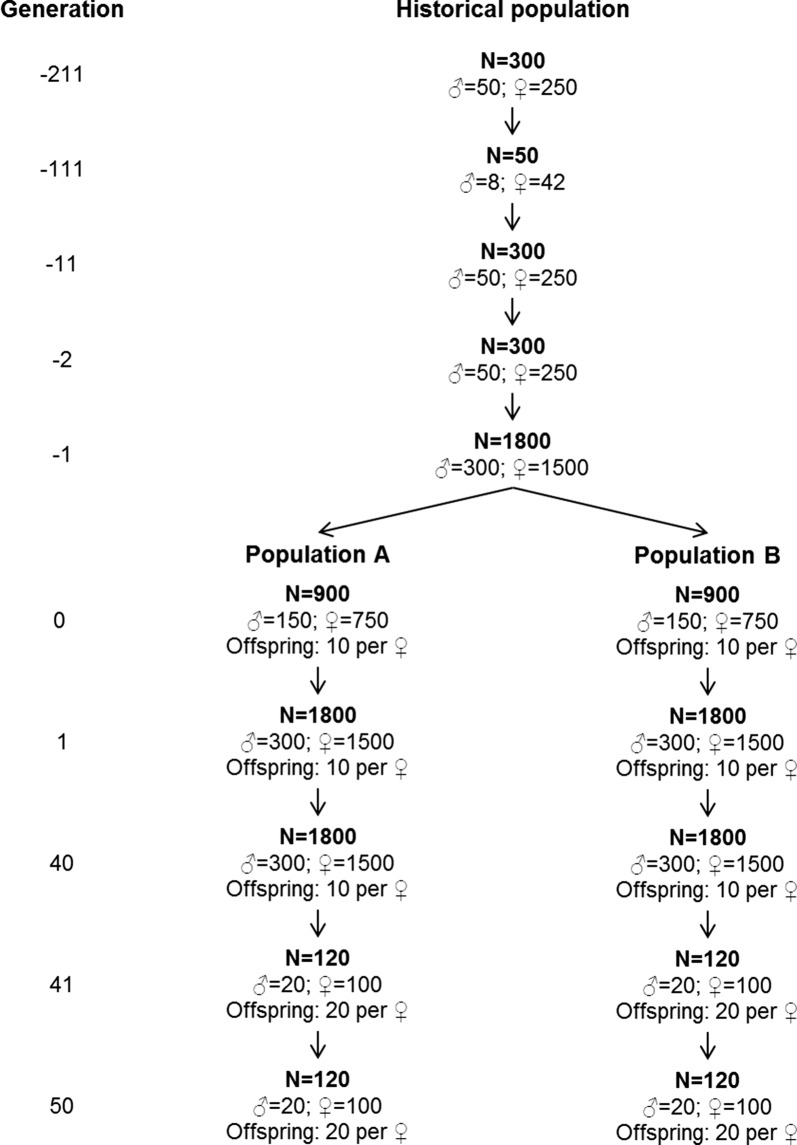
Schematic representation of the simulated population structure

Two populations were simulated using the QMSim software [24]. The simulations were set-up with two characteristics: (1) the two populations have different LD patterns, as measured by the LD statistic $r$; and (2) a large number of loci segregate in the last generation with part of these (> 200,000) having similar allele frequencies in both populations, and another part (> 200,000) having different allele frequencies in each population. We simulated a historical population for 211 generations (Fig. 1). The first generation (generation − 211) contained 300 individuals. During the following 100 generations (generations − 211 to − 112), population size decreased gradually to 50 individuals to create LD. From generation -111 to generation − 12, population size increased gradually to 300 individuals and was kept constant for the next 10 generations (generations − 11 to − 2). In the last generation of the historical population (generation − 1), population size increased to 1800 individuals. In the entire historical population, generations were discrete, there was no selection, mating was at random, and the male to female ratio was 1:5. The effective population size ($N_{e}$) of the entire historical population was ~ 79, which was approximated as the harmonic mean of $N_{e}$ in each generation calculated as: $\frac{1}{{N_{e} }} = \frac{1}{{4N_{m} }} + \frac{1}{{4N_{f} }}$, where $N_{m}$ is the number of males and $N_{f}$ the number of females used to create the next generation [25].

The last generation of the historical population was divided randomly into two equally-sized populations ($A$ and $B$) of 900 individuals. In the next generation, the size of both populations was increased to 1800 individuals and was kept constant for the following 40 generations (generations 1–40). These reasonably large population sizes limited the drift of allele frequencies. The number of offspring was set to 10 and selection was at random, such that the number of selected offspring per individual followed approximately a Poisson distribution, as assumed in the Wright-Fisher model of genetic drift. During the last 10 generations (generations 41–50), population size decreased to 120 individuals in each population to increase the extent of LD in each population, and the number of offspring was set to 20. Similar to the historical population, these generations were discrete, there was no selection, mating was at random, and the male to female ratio was 1:5. All individuals from the last generation (2000) were used for the analyses.

### Genome size

A genome of 10 chromosomes of one Morgan each was simulated. This genome size was a balance between the computational effort necessary for the analyses and the variation in relationships between family members [26]. Each chromosome contained 300,000 randomly spaced loci, with a recurrent mutation rate of 0.00005 in the historical population. In the last generation of the historical population, segregating loci were selected and mutation was stopped. The chosen population size and mutation rate resulted in a U-shaped allele frequency distribution of segregating loci in the two populations, as common in real populations.

In the last generation (generation 50), markers and 2000 causal loci were selected from all segregating loci. Three marker panels were constructed: a high-density panel (HDP) with 200,000 markers, a medium-density panel (MDP) with 20,000 markers, and a low-density panel (LDP) with 2000 markers. Each of the smaller marker panels was a subset from the larger marker panels.

Markers and causal loci were selected to have either similar or different allele frequencies in populations $A$ and $B$. For both approaches, three selection criteria were used; namely (1) the segregation in one or both populations, (2) the absolute difference in allele frequency between population $A$ ($p_{A}$) and population $B$ ($p_{B}$), and (3) the difference in $2p\left( {1 - p} \right)$ between populations $A$ and $B$, which is a measure of the difference in variance explained by a locus when allele substitution effects are the same in the two populations. The last criterion was mainly effective for loci with a low allele frequency, since an apparently small difference in allele frequency can result in a relatively large difference in variance explained for those loci. This criterion was used to ensure that the proportion of genetic variance explained by a locus was more or less similar in the two populations when populations had similar allele frequencies.

As a first step, markers were selected from the segregating loci. To select markers with similar allele frequencies in the two populations, (1) loci had to segregate in both populations, (2) $\left| {p_{A} - p_{B} } \right|$ should be less than 0.14, and (3) $\left| {\left[ {2p_{A} \left( {1 - p_{A} } \right) - 2p_{B} \left( {1 - p_{B} } \right)} \right]} \right|/\left[ {2\bar{p}_{AB} \left( {1 - \bar{p}_{AB} } \right)} \right]$ should be less than 2, where $\bar{p}_{AB}$ is the average of $p_{A}$ and $p_{B}$. To select markers with different allele frequencies in the two populations, (1) loci had to segregate in at least one population, (2) $\left| {p_{A} - p_{B} } \right|$ should be more than 0.14, and (3) $\left| {\left[ {2p_{A} \left( {1 - p_{A} } \right) - 2p_{B} \left( {1 - p_{B} } \right)} \right]} \right|/\left[ {2\bar{p}_{AB} \left( {1 - \bar{p}_{AB} } \right)} \right]$ should be more than 1. The cut-off values were chosen to either minimize or maximize the difference in allele frequencies between the populations, while ensuring that enough loci in each replicate met the criteria. Our aim was to select marker panels with a uniform distribution of allele frequencies to reflect commercially available marker chips [27–30]. For this step, the loci that met the criteria were divided into 50 bins based on average allele frequency over the two populations (i.e., allele frequencies of bin 1 ranged from 0 to 0.02, of bin 2 from 0.02 to 0.04, etc.), and from each bin an equal number of loci was randomly selected. When the number of loci was too small in the two extreme bins (0.00–0.02, and 0.98–1.00), the bins were combined with the neighbouring bin.

As a second step, causal loci were selected from the segregating loci that were not selected as markers. To select causal loci, the same criteria and cut-off values were used as for markers, with one exception. In all scenarios, causal loci did not have to segregate in both populations, since some causal loci are known to be at least partly population-specific [31]. Causal loci were selected randomly from all simulated loci that met the criteria, and therefore the pattern of their allele frequencies followed an approximate U-shaped distribution as expected for causal loci [32, 33].

### LD patterns and consistency of LD between populations

The LD pattern and consistency of LD between the populations was investigated. Within each population and between all causal loci and markers less than 10 cM apart, the parameter $r$ was calculated [34]:

$$r = \frac{{\left( {f_{11} f_{22} - f_{12} f_{21} } \right)}}{{\sqrt {f_{1.} f_{2.} f_{.1} f_{.2} } }},$$

where $f_{11}$ is the haplotype frequency with allele 1 at the first locus and allele 1 at the second locus, $f_{22}$, $f_{12}$ and $f_{21}$ are frequencies of the other possible haplotypes, $f_{1.}$ and $f_{2.}$ are the frequencies of allele 1 and allele 2 at the first locus, and $f_{.1}$ and $f_{.2}$ are the frequencies of allele 1 and allele 2 at the second locus. The LD pattern within each population was represented by the average $r^{2}$ for intervals of 0.1 cM between the markers. The consistency of LD between the two simulated populations $A$ and $B$ was calculated as the correlation between $r$ values of the two populations for intervals of 0.1 cM, following De Roos et al. [35]. The consistency of LD between populations is known to decrease when the genomic distance between markers and causal loci increases [35]. This indicates that the populations were expected to have a higher consistency of LD between markers and causal loci when using HDP markers than when using MDP or LDP markers. In this way, the analysis with the HDP markers represented a situation with the highest consistency of LD between populations, and the analysis with LDP markers represented a situation with the lowest consistency of LD between populations.

### Phenotypes

For each causal locus, allele substitution effects were sampled from a bivariate normal distribution, with a mean of 0, a standard deviation of 1, and a correlation between the populations of 1, 0.8, 0.6, 0.4, 0.2 or 0. For each individual, its allele counts for the causal loci (coded as 0, 1, and 2) were multiplied by the corresponding allele substitution effects and the results were summed over loci to calculate the additive genetic value (AGV) of the individual. The AGV were scaled to a mean of 0 and a variance of 1 across all individuals. Since allele substitution effects were sampled independently from allele frequency, the correlation between AGV of populations $A$ and $B$ (i.e., genetic correlation) was similar to the correlation between allele substitution effects (i.e., 1, 0.8, 0.6, 0.4, 0.2 or 0). A normally distributed environmental effect was sampled for each individual to obtain a heritability of 0.3 in each population. Phenotypes of all 2000 individuals in generation 50 were computed by summing the AGV and the environmental effects.

The simulation of phenotypes was replicated 50 times for each scenario. For each replicate, markers and causal loci were selected for three scenarios; (1) similar allele frequencies between populations for both markers and causal loci, (2) similar allele frequencies between populations for markers and different allele frequencies between populations for causal loci, and (3) different allele frequencies for both markers and causal loci. Within each scenario, phenotypes were simulated for each of the six genetic correlations. Scripts and seeds to simulate the data are in Additional file 2.

### Estimation of the genetic correlation

The additive genetic correlation between populations was estimated using the following bivariate model:

$$\left[ {\begin{array}{*{20}c} {{\mathbf{y}}_{A} } \\ {{\mathbf{y}}_{B} } \\ \end{array} } \right] = \left[ {\begin{array}{*{20}c} {{\mathbf{x}}_{A} } & {\bf 0} \\ {\bf 0} & {{\mathbf{x}}_{B} } \\ \end{array} } \right]\left[ {\begin{array}{*{20}c} {\mu_{A} } \\ {\mu_{B} } \\ \end{array} } \right] + \left[ {\begin{array}{*{20}c} {{\mathbf{Z}}_{A} } & {\bf 0} \\ {\bf 0} & {{\mathbf{Z}}_{B} } \\ \end{array} } \right]\left[ {\begin{array}{*{20}c} {{\mathbf{a}}_{A} } \\ {{\mathbf{a}}_{B} } \\ \end{array} } \right] + \left[ {\begin{array}{*{20}c} {{\mathbf{e}}_{A} } \\ {{\mathbf{e}}_{B} } \\ \end{array} } \right],$$

where ${\mathbf{y}}_{k}$ is a vector of phenotypes for population $k$ ($k = A, B$*)*, ${\mathbf{x}}_{k}$ is an incidence vector relating phenotypes to the mean in population $k$ ($\mu_{k}$), ${\mathbf{Z}}_{k}$ is an incidence matrix relating phenotypes to estimated additive genetic values $$\left( {\left[ {\begin{array}{*{20}c} {{\mathbf{a}}_{A} } \\ {{\mathbf{a}}_{B} } \\ \end{array} } \right] \sim N\left( {\left[ {\begin{array}{*{20}c} {\bf 0} \\ {\bf 0} \\ \end{array} } \right],\left[ {\begin{array}{*{20}c} {\sigma_{A}^{2} } & {\sigma_{AB} } \\ {\sigma_{AB} } & {\sigma_{B}^{2} } \\ \end{array} } \right] \otimes \left[ {\begin{array}{*{20}c} {{\mathbf{G}}_{AA} } & {{\mathbf{G}}_{AB} } \\ {{\mathbf{G}}_{BA} } & {{\mathbf{G}}_{BB} } \\ \end{array} } \right]} \right)} \right)$$ with $\otimes$ representing the Kronecker product function, and ${\mathbf{e}}_{k}$ are vectors with independent residual effects. Genetic and residual variances were estimated using restricted maximum likelihood (REML). The first analyses were performed using the ASReml software [36]. For the scenarios analysed later, we switched to MTG2 [37] to reduce computation time. We verified that the estimated variance components were identical using both programs.

The genomic relationship matrix (${\mathbf{G}}$) between all individuals was calculated as [17]:

$$\begin{aligned} {\mathbf{G}} & = \left[ {\begin{array}{*{20}c} {{\mathbf{G}}_{AA} } & {{\mathbf{G}}_{AB} } \\ {{\mathbf{G}}_{BA} } & {{\mathbf{G}}_{BB} } \\ \end{array} } \right] \\ & = \left[ {\begin{array}{*{20}c} {\frac{{{\mathbf{W}}_{A} {\mathbf{W}}_{A}^{\prime } }}{{\sum 2p_{Ai} \left( {1 - p_{Ai} } \right)}}} & {\frac{{{\mathbf{W}}_{A} {\mathbf{W}}_{B}^{\prime } }}{{\sqrt {\sum 2p_{Ai} \left( {1 - p_{Ai} } \right)} \sqrt {\sum 2p_{Bi} \left( {1 - p_{Bi} } \right)} }}} \\ {\frac{{{\mathbf{W}}_{B} {\mathbf{W}}_{A}^{\prime } }}{{\sqrt {\sum 2p_{Ai} \left( {1 - p_{Ai} } \right)} \sqrt {\sum 2p_{Bi} \left( {1 - p_{Bi} } \right)} }}} & {\frac{{{\mathbf{W}}_{B} {\mathbf{W}}_{B}^{\prime } }}{{\sum 2p_{Bi} \left( {1 - p_{Bi} } \right)}}} \\ \end{array} } \right], \\ \end{aligned}$$

where ${\mathbf{W}}_{k}$ is a matrix with centered allele counts of all individuals from population $k$, and $p_{ki}$ is the allele frequency for locus $i$ in population $k$. Centered allele counts were calculated as $g_{ijk} - 2p_{ki}$, where $g_{ijk}$ is the allele count of locus $i$ for individual $j$ from population $k$, coded as 0, 1 or 2. This ${\mathbf{G}}$ defines the relationships as standardized covariances between the genetic values of individuals [17]. In all scenarios and for all replicates, we calculated ${\mathbf{G}}$ using allele counts of (1) causal loci, (2) HDP markers, (3) MDP markers, or (4) LDP markers in both populations.

The relationships at causal loci are the true relationships for that trait, which are approximated when using markers. Marker-based relationships are subject to sampling error, since markers are a subset of the genome and in imperfect LD with the causal loci. A way to account for this sampling error is by regressing ${\mathbf{G}}$ towards the pedigree relationship matrix (${\mathbf{A}}$) [32, 38, 39], which is expected to reduce bias of the estimated variance components [32]. To investigate the effect of this regression, ${\mathbf{G}}$ matrices based on the three marker panels were regressed towards ${\mathbf{A}}$ and used for the scenarios with a correlation of 0.8 or 0.4.

Before regressing ${\mathbf{G}}$ towards ${\mathbf{A}}$, the inbreeding level of each within-population block in ${\mathbf{G}}$ was rescaled to the inbreeding level in ${\mathbf{A}}$, following [39]:

$${\mathbf{G}}^{*} = \left( {1 - \bar{F}_{k} } \right){\mathbf{G}} + 2\bar{F}_{k} {\mathbf{J}},$$

where $\bar{F}_{k}$ is the average inbreeding coefficient of all individuals of population $k$ based on the pedigree, and ${\mathbf{J}}$ is a matrix of 1s. The rescaled ${\mathbf{G}}^{*}$ was regressed towards ${\mathbf{A}}$ following [32, 38]:

$${\hat{\mathbf{G}}} = {\mathbf{A}} + b\left( {{\mathbf{G}}^{*} - {\mathbf{A}}} \right)\quad {\text{with}}\quad b = \frac{{Var\left( {{\mathbf{G}}^{*} - {\mathbf{A}}} \right)}}{{Var\left( {{\mathbf{G}}^{*} - {\mathbf{A}}} \right) + \frac{1}{n}}},$$

where $n$ is the number of markers. To set-up ${\mathbf{A}}$, the pedigree of the last 10 generations was used, such that between-population ${\mathbf{A}}$ relationships were zero. The regression was done separately within each population per bin of pedigree relationships (< 0.10, 0.10–0.25, 0.25–0.50, > 0.5) and between populations, since regression coefficients are higher for stronger pedigree relationships [40, 41]. For the diagonal elements, only the inbreeding coefficients in ${\mathbf{G}}^{*}$ were regressed towards ${\mathbf{A}}$ [32]. Regression coefficients were all close to 1 for higher marker density panels (> 0.99 for HDP and > 0.97 for MDP). For LDP markers, regression coefficients were lower, i.e. ~ 0.84 for between-population relationships, ~ 0.89, ~ 0.91, ~ 0.94 and ~ 0.96 for the four bins of within-population relationships, and ~ 0.93 for inbreeding coefficients.

## Results

### Characteristics of the simulations

**Fig. 2:**
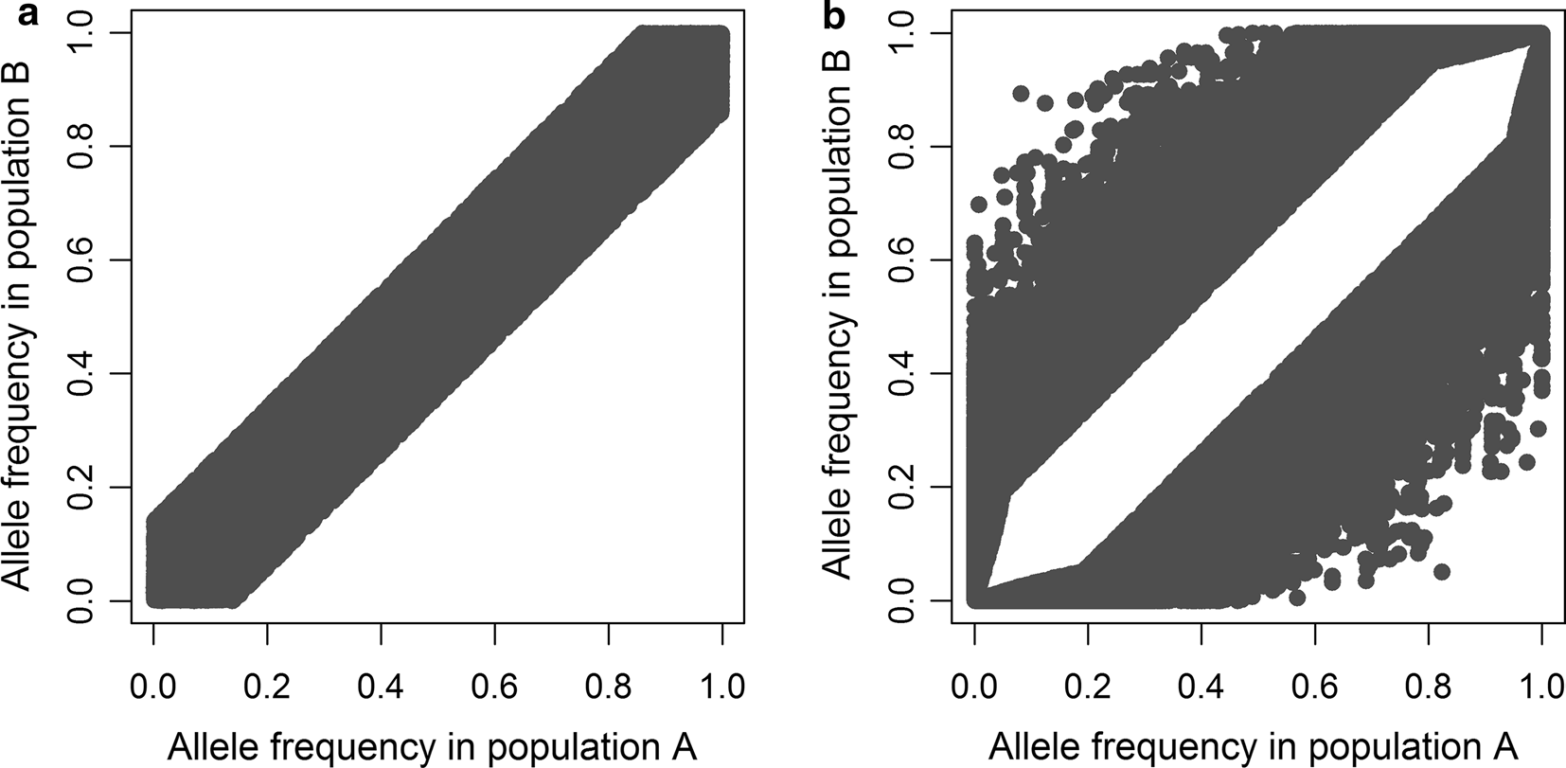
Allele frequencies of markers in two populations using two approaches to select markers. For one random replicate, allele frequencies of markers from both populations are plotted against each other for markers that are selected to have **a** similar allele frequencies or **b** different allele frequencies in the two populations

**Fig. 3:**
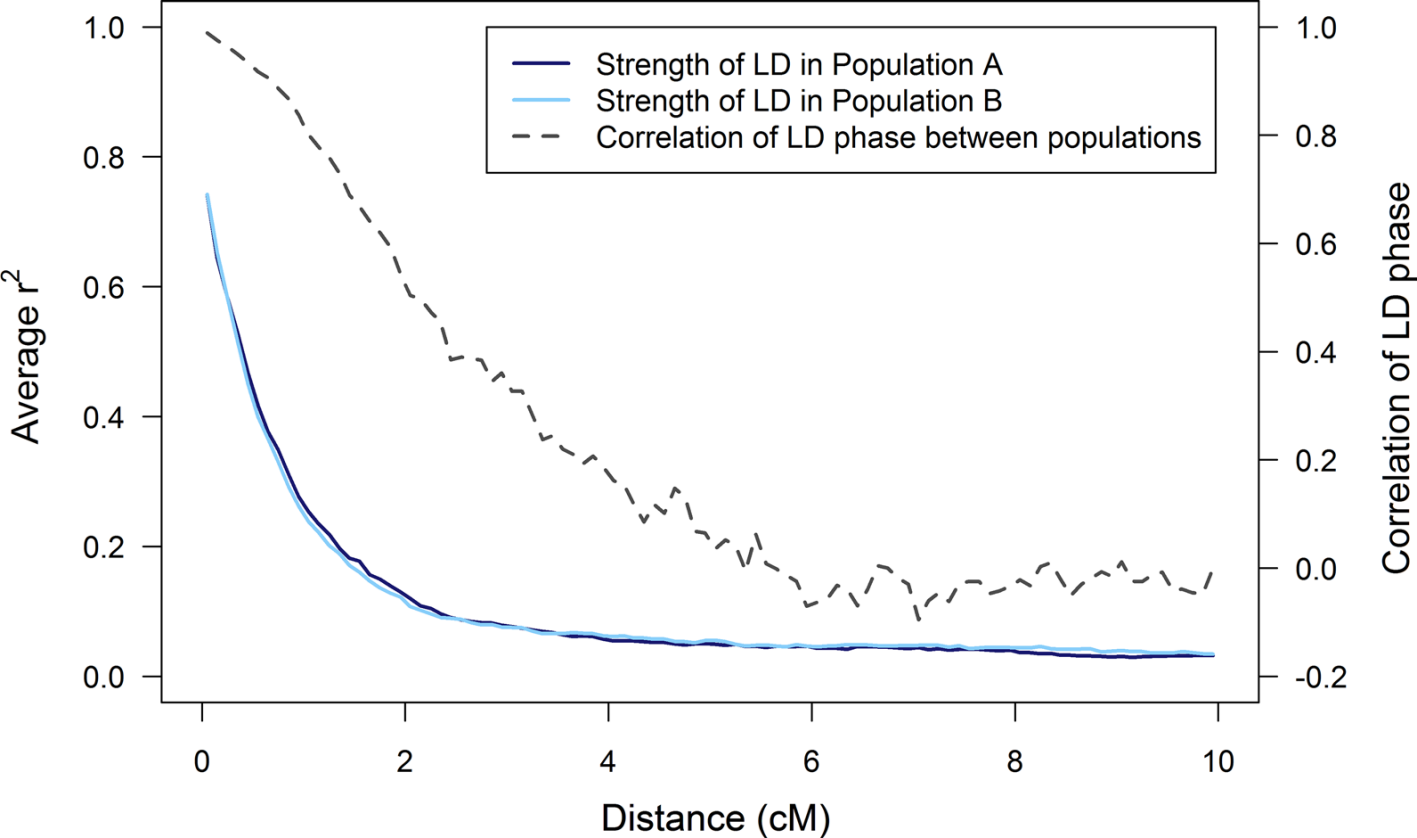
Extent of LD in two populations and correlation of LD phase between the populations as a function of distance. The average LD ($r^{2}$) between causal loci and markers for both populations, and the correlation of LD-phase (correlation of $r$) between the populations, as a function of distance between causal loci and markers for one random replicate

The criteria for selecting markers and causal loci resulted in clear differences between the scenarios with similar and different allele frequencies in the two populations (Fig. 2) and [see Additional file 1]. As designed in the simulations, the distribution of allele frequencies was uniform for markers and U-shaped for causal loci as expected in livestock populations. Therefore, the percentage of causal loci with a minor allele frequency lower than 0.05 was higher (on average 33% in each population) than the percentage of markers with a minor allele frequency lower than 0.05 (on average only 15% in each population). The decay of LD was similar in both populations (Fig. 3), with a stronger decay as distances between the loci in the 0–2 cM interval increased. The correlation of LD phase between the populations, representing the consistency of LD phase between the populations, decreased rapidly at short distances (0–5 cM), and fluctuated around zero at distances longer than 5 cM. As designed in the simulations, the extent and consistency of LD between populations were comparable to those in chicken and pig populations [20, 42–44].

### Proportion of explained variance

The proportion of the phenotypic variance explained by the markers, known as the genomic heritability [45], was close to the simulated heritability for the scenarios with HDP and MDP markers and slightly lower than the simulated heritability for the scenarios with LDP markers (estimated: ~ 0.29; simulated: 0.3). This implies that genetic variances were estimated accurately regardless of the marker panel used.

### Estimated genetic correlation

**Fig. 4:**
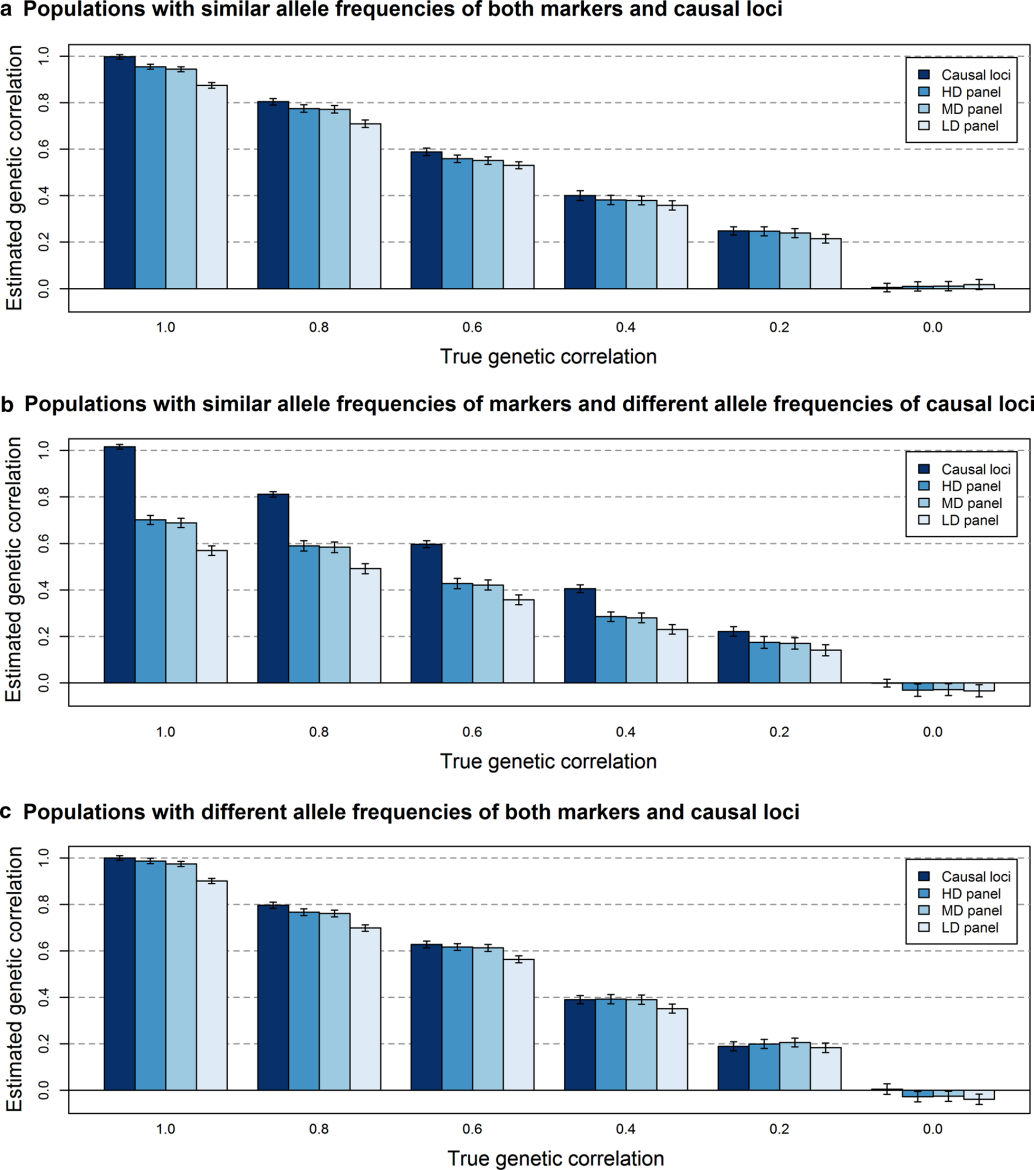
Estimated genetic correlations between populations without regressing the genomic relationship matrix. The average estimated genetic correlation (± standard error of the mean) at different simulated genetic correlations for the scenario in which **a** markers and causal loci have similar allele frequencies in the two populations, **b** markers have similar and causal loci different allele frequencies in the two populations, or **c** markers and causal loci have different allele frequencies in the two populations, when the genomic relationship matrix is either based on the genotypes of causal loci (2000), HDP (200,000), MDP (20,000), or LDP (2000) markers without regression towards the pedigree relationship matrix. Standard errors were calculated as the standard deviation over replicates divided by the square root of the number of replicates

With relationships based on causal loci, all estimated genetic correlations were unbiased, irrespective of whether causal loci had similar or different allele frequencies in the two populations (Fig. 4). This was expected based on our previous results [17].

With relationships based on markers, all estimated genetic correlations were slightly to severely biased. The bias was very small when the difference in allele frequencies between the two populations was similar for the markers and the causal loci. For example, when marker-based relationships were not regressed towards the pedigree relationships, genetic correlations were underestimated by only ~ 2.5% for HDP, ~ 3% for MDP, and ~ 11% for LDP markers (Fig. 4a, c). The bias was much larger when markers had similar allele frequencies in the two populations and causal loci had different allele frequencies, i.e. when the difference in allele frequencies between the two populations differed between markers and causal loci (Fig. 4b; ~ 28% for HDP, ~ 30% for MDP, and ~ 41% for LDP markers). It should be noted that the distribution of allele frequencies was always uniform for markers and always U-shaped for causal loci.

**Fig. 5:**
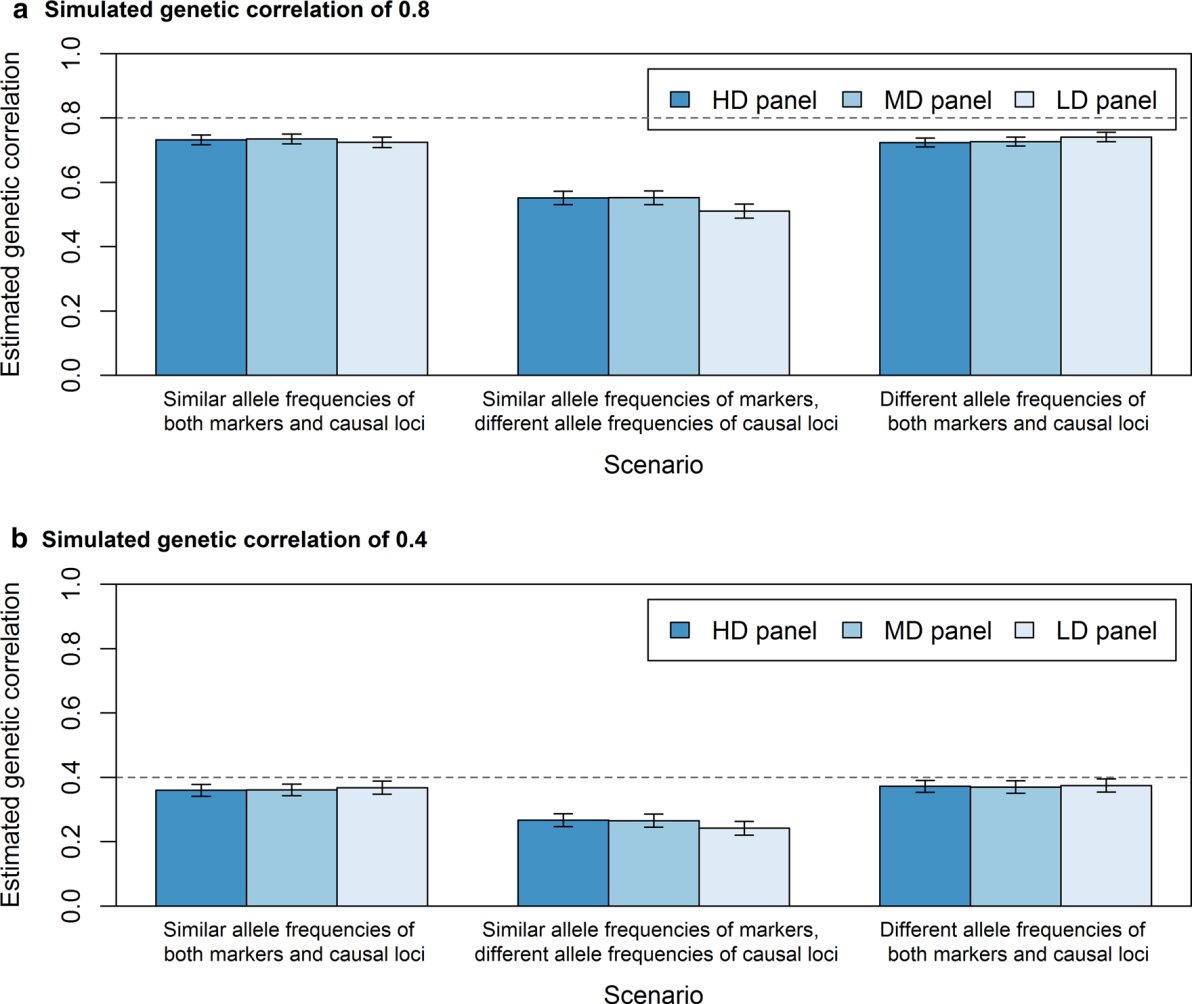
Estimated genetic correlations between populations with regression of the genomic relationship matrix. The average estimated genetic correlation (± standard error of the mean) at a simulated genetic correlation of **a** 0.8 or **b** 0.4 for the three scenarios with HDP (200,000), MDP (20,000), or LDP (2000) markers and regression of ${\mathbf{G}}$ towards ${\mathbf{A}}$. Standard errors were calculated as the standard deviation over replicates divided by the square root of the number of replicates

Across all scenarios, regressing ${\mathbf{G}}$ towards ${\mathbf{A}}$ had only a small effect on the estimated genetic correlation (Fig. 5). At a high marker density, regressing ${\mathbf{G}}$ towards ${\mathbf{A}}$ lowered the estimated genetic correlation. Therefore, the underestimation for HDP and MDP markers increased from ~ 4 to ~ 9% when the difference in allele frequencies between the populations was similar for markers and causal loci, and from ~ 28 to ~ 32% when this was not the case. In contrast, regressing ${\mathbf{G}}$ towards ${\mathbf{A}}$ resulted in higher estimated genetic correlations at low marker density. For LDP markers, the underestimation decreased from ~ 12 to ~ 8% when the difference in allele frequencies between the populations was similar for markers and causal loci and from ~ 41 to ~ 38% when this was not the case. Thus, regressing ${\mathbf{G}}$ towards ${\mathbf{A}}$ was only beneficial for the estimation of the genetic correlation between populations when the marker density was low.

In general, standard errors of the mean across replicates for the estimated genetic correlation were small for all scenarios (~ 0.02), and tended to be slightly larger for lower true genetic correlations. Moreover, standard errors were slightly larger when the difference in allele frequencies between populations was not similar for markers and causal loci (Fig. 4b vs a, c). Regression of ${\mathbf{G}}$ towards ${\mathbf{A}}$ had no effect on the standard error.

### Genomic relationships

**Fig. 6:**
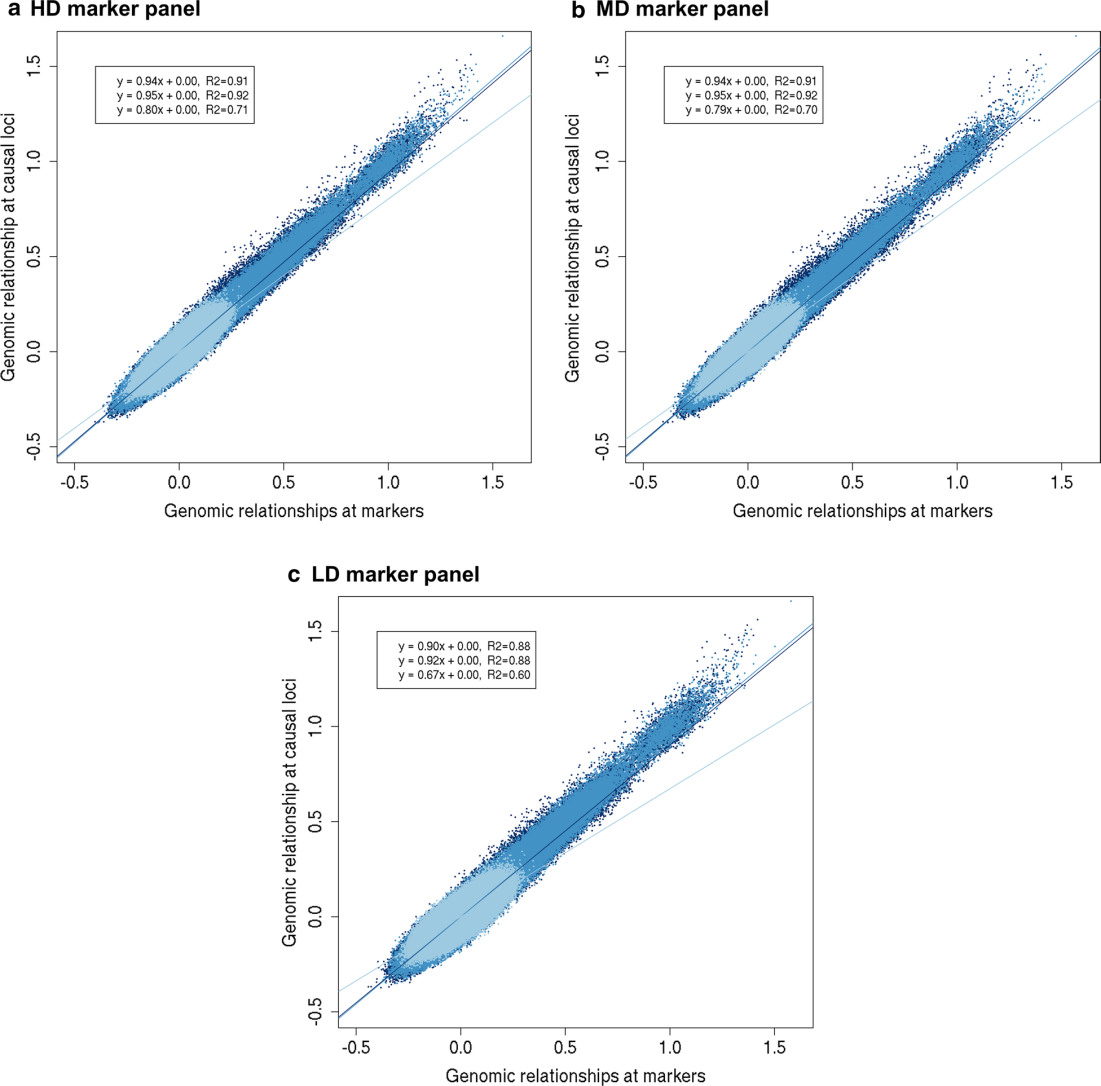
Genomic relationships at causal loci versus markers when causal loci have similar allele frequencies in the two populations. The genomic relationships at the causal loci versus the genomic relationships based on **a** HDP (200,000) markers, **b** MDP (20,000) markers, or **c** LDP (2000) markers, when markers and causal loci have similar allele frequencies in the two populations for one replicate. Relationships in population ${\text{A}}$ are represented in dark blue (Eq. 1 of regression line and correlation), relationships in population ${\text{B}}$ are represented in medium blue (Eq. 2 of regression line and correlation), and relationships between population ${\text{A}}$ and ${\text{B}}$ are represented in light blue (Eq. 3 of regression line and correlation)

**Fig. 7:**
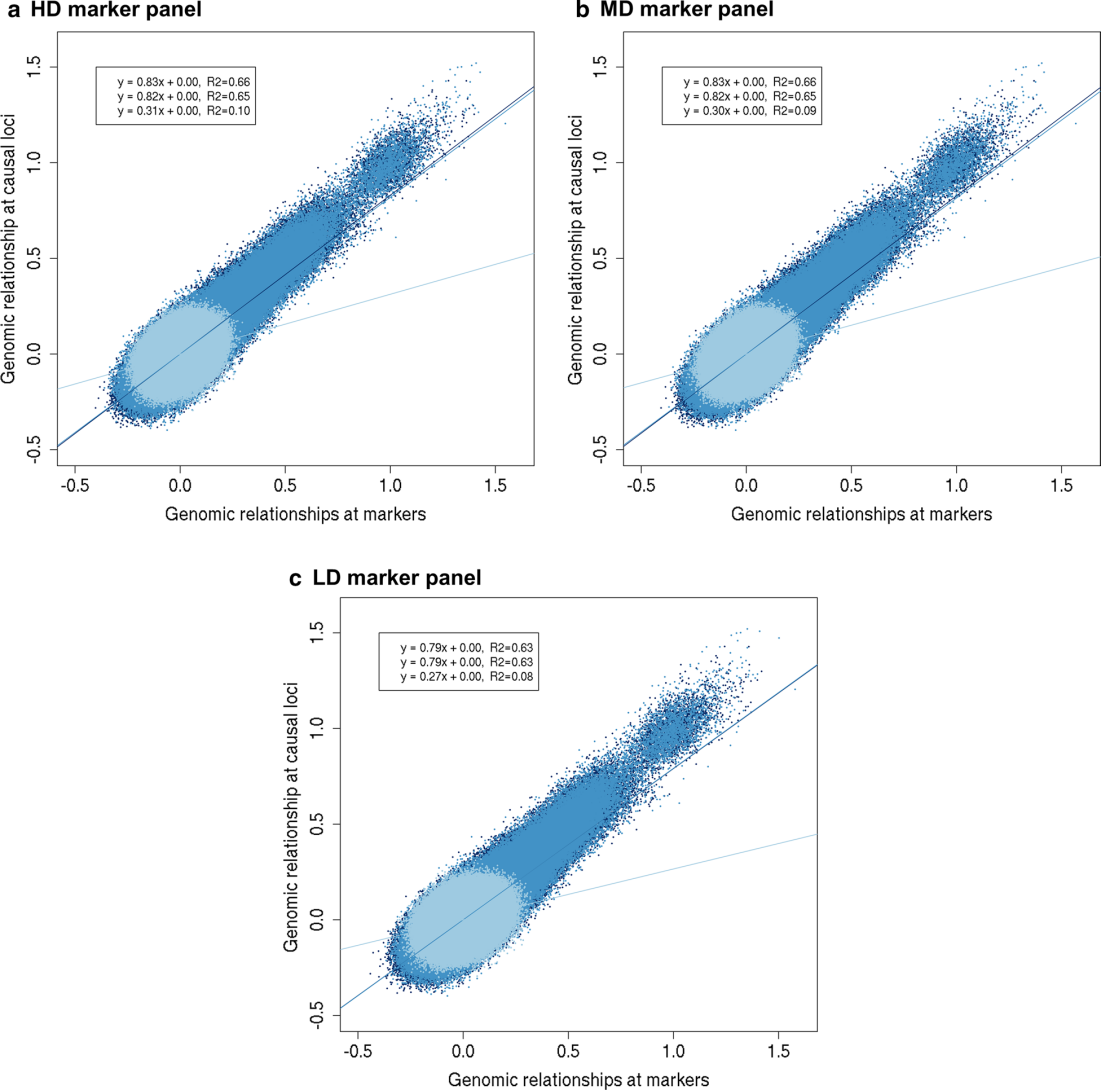
Genomic relationships at causal loci versus markers when causal loci have different allele frequencies in the two populations. The genomic relationships at the causal loci versus the genomic relationships based on the **a** HDP (200,000) markers, **b** MDP (20,000) markers, or **c** LDP (2000) markers, when markers have similar and causal loci different allele frequencies in the two populations for one replicate. Relationships in population ${\text{A}}$ are represented in dark blue (Eq. 1 of regression line and correlation), relationships in population ${\text{B}}$ are represented in medium blue (Eq. 2 of regression line and correlation), and relationships between population ${\text{A}}$ and ${\text{B}}$ are represented in light blue (Eq. 3 of regression line and correlation)

Estimates of the genetic variance are biased when the regression of true relationships on marker-based relationships is not equal to 1 [38]. We investigated whether this could explain the underestimation of the genetic correlation by considering the genomic relationships at the causal loci as the true relationships for that trait. In Figs. 6 and 7, we plotted the relationships at the causal loci versus the unregressed relationships at the markers for one of the replicates. The regression coefficients for within-population genomic relationships were close to 1, and were only slightly lower when causal loci had different allele frequencies (Fig. 7) compared to similar allele frequencies (Fig. 6) in the two populations. This means that the within-population relationships at the markers can predict quite accurately the relationships at the causal loci.

Regression coefficients of between-population relationships deviated more from 1, especially at low marker density. When the difference in allele frequencies between the populations was similar for markers and causal loci, the regression coefficients were equal to ~ 0.8 for HDP and MDP and 0.67 for LDP markers (Fig. 6). This means that the relationships at the markers led to an over-prediction of the relationships at the causal loci. When the difference in allele frequencies between the populations was not similar for markers and causal loci, regression coefficients of between-population relationships were equal to ~ 0.30 (Fig. 7). Thus, the over-prediction of between-population relationships using markers was much larger when the difference in allele frequencies between the populations was not similar for markers and causal loci.

The correlation between the relationships at the causal loci and at the markers, i.e., the accuracy of the marker-based relationships, decreased when the density of the markers decreased (Figs. 6, 7). When the difference in allele frequencies between the populations was similar for markers and causal loci, the correlation for within-population relationships was ~ 0.91 for HDP and MDP, and ~ 0.88 for LDP markers. The correlation for between-population relationships was ~ 0.70 for HDP and MDP, and 0.60 for LDP markers. The correlation between relationships at causal loci and at markers was much lower when the difference in allele frequencies between the populations was not similar for markers and causal loci (within-population relationships: ~ 0.66 for HDP and MDP, ~ 0.63 for LDP; between-population relationships: ~ 0.09 for HDP and MDP, ~ 0.08 for LDP).

## Discussion

Our objective was to investigate whether differences in LD patterns between populations and differences in allele frequencies of markers and/or causal loci between populations affect bias of the estimated genetic correlation. We simulated two populations that differed in LD pattern between markers and causal loci, as measured by the LD-statistic $r$. Our results show that when the difference in allele frequencies between the two populations is similar for markers and causal loci, estimated genetic correlations are only slightly underestimated using markers. When the difference in allele frequencies between the two populations was not similar for markers and causal loci, genetic correlations were severely underestimated. Differences in LD and allele frequencies of causal loci between populations had only a very slight effect on the precision of the estimated genetic correlation.

### Estimation of genetic correlations using marker-based relationships

Estimates of the genetic variance and heritability are known to be biased when the regression coefficient of the true relationships on the marker-based relationships is not equal to 1, i.e., when $E\left( {{\mathbf{G}}_{causal\:loci} |{\mathbf{G}}_{markers} } \right) \ne {\mathbf{G}}_{markers}$ [32, 38, 46]. When this regression coefficient is less than 1, relationships at the markers show too much variation, which results in an underestimation of the genetic variance. Yang et al. [32] argued that a regression coefficient less than 1 can be due to two effects: (1) sampling error on the relationships because the number of markers is finite; and (2) a difference in the distribution of allele frequencies between causal loci and markers. In all our scenarios, the number of markers was finite and the distribution of allele frequencies differed between causal loci and markers. However, within populations, the estimated genomic heritability [45] was close to the simulated trait heritability for all scenarios. This suggests that the number of markers used was sufficient to constrain the sampling error on within-population relationships to an acceptable level, and that our estimated genetic variances were affected only slightly by the difference in the distribution of allele frequencies between causal loci and markers. Thus, the underestimation of the genetic correlation between populations is not a result of biased estimates of the genetic variance.

The relative sampling error due to the use of a finite number of markers is much larger for between-population relationships than for within-population relationships, because more markers are needed to accurately estimate the small between-population relationships [38]. Moreover, we showed that the accuracy of predicting the between-population relationships at the causal loci using markers depended on whether the difference in allele frequency of causal loci between populations was reflected by the markers. These two factors can result in an underestimation of the genetic covariance between populations, which can explain the slight underestimation of the genetic correlation in the scenarios in which the difference in allele frequencies between the two populations was similar for markers and causal loci and the larger underestimation in the scenarios in which this was not the case. The higher sampling error on between-population relationships can also explain the larger underestimation of the genetic correlation for the LDP markers than for the HDP and MDP markers. Thus, to estimate the genetic correlation between populations, it is important that the difference in allele frequencies between the populations is similar for markers and causal loci and that the number of markers is sufficiently large.

The additive genetic correlation between populations is defined as the correlation between the two additive genetic values of a single genotype, measured as the allele count at each causal locus, in the two populations. This correlation is equal to the correlation between allele substitution effects when allele substitution effects are independent of allele frequency. This independency was used in our simulated phenotypes and also to set-up the ${\mathbf{G}}$ matrix, as it is an implicit assumption in Method 1 of VanRaden [47]. When this assumption is not met, the genetic correlation is no longer equal to the correlation between allele substitution effects, and violation of this assumption in the set-up of ${\mathbf{G}}$ may result in biased estimates of the genetic correlation.

### Regression of the maker-based relationships

Regressing ${\mathbf{G}}$ towards ${\mathbf{A}}$ is a way of correcting the marker-based relationships for the sampling error due to a finite number of markers [39]. The regression was strongest for LDP markers and reduced the underestimation of the genetic correlation. These results agree with the findings that regressing ${\mathbf{G}}$ towards ${\mathbf{A}}$ is important when the number of markers is small [32] and supports our statement that relationships at LDP markers were affected by sampling error. However, regressing ${\mathbf{G}}$ towards ${\mathbf{A}}$ slightly increased the underestimation of the genetic correlation with HDP and MDP markers. The reason for this is not clear. It might be that the regression of ${\mathbf{G}}$ towards ${\mathbf{A}}$ not only reduces the sampling error, but also amplifies the effect of the difference in the distribution of allele frequencies between causal loci and markers.

In our study, regressing ${\mathbf{G}}$ towards ${\mathbf{A}}$ was slightly detrimental for the estimation of the genetic correlation when using HDP (200,000) or MDP (20,000) markers, with regression coefficients being only slightly less than 1, and it was beneficial when using LDP (2000) markers with regression coefficients being considerably less than 1. The simulated genome represented about one-third of the genome of livestock species such as cattle and chicken [48, 49]. This suggests that regressing ${\mathbf{G}}$ could be detrimental when using a genome-wide total of 60,000 or more markers in livestock. Note that this number of markers will depend on the consistency of LD between populations. Between-population relationships are all closer to zero when the LD pattern is less consistent between populations [50]. Such weaker relationships generally require more markers to reduce their relative sampling error to an acceptable level [32]. Hence, we think that the regression coefficients may be a better indicator for deciding whether or not to regress ${\mathbf{G}}$; when all regression coefficients are close to 1, e.g., higher than 0.95, it is probably better not to regress ${\mathbf{G}}$ towards ${\mathbf{A}}$ when estimating the genetic correlation between populations.

**Fig. 8:**
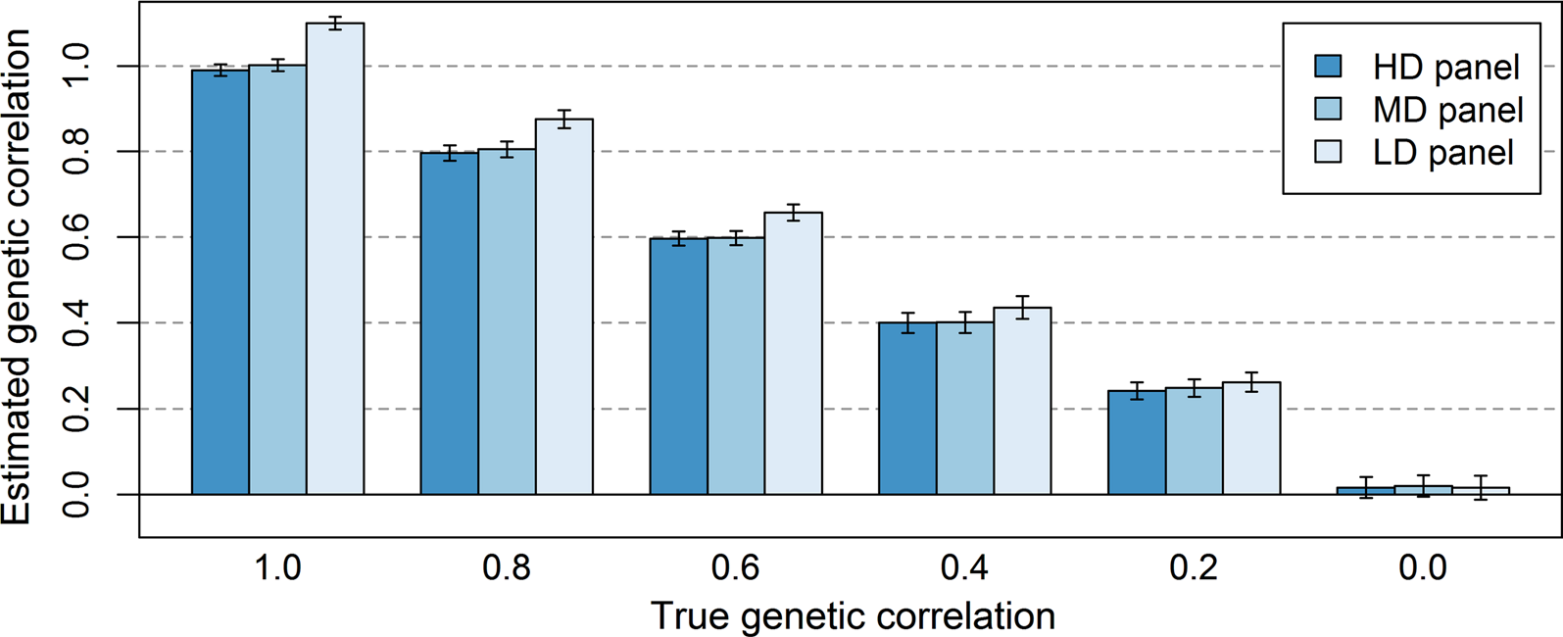
Estimated genetic correlations between populations after rescaling the marker-based genomic relationship matrix. The average estimated genetic correlation (± standard error of the mean) at different simulated genetic correlations for the scenario in which markers and causal loci have similar allele frequencies in the two populations when the genomic relationship matrix is either based on the genotypes of HDP (200,000), MDP (20,000), or LDP (2000) markers, after rescaling the marker-based relationships using a regression coefficient based on the relationships at causal loci. Standard errors were calculated as the standard deviation over replicates divided by the square root of the number of replicates

The coefficients to regress ${\mathbf{G}}$ towards ${\mathbf{A}}$ were approximated using the number of markers and the variation in ${\mathbf{G}}_{markers} - {\mathbf{A}}$, assuming that the sampling error was only a result of using a limited number of markers [38]. To investigate the impact of this approximation, we repeated the analysis by using $b = \frac{{Cov\left( {{\mathbf{G}}_{causal\:loci} - {\mathbf{A}}, {\mathbf{G}}_{markers} - {\mathbf{A}}} \right)}}{{Var\left( {{\mathbf{G}}_{markers} - {\mathbf{A}}} \right)}}$ [38] as the regression coefficient to regress ${\mathbf{G}}$ towards ${\mathbf{A}}$. This regression requires that the causal loci are known, which generally is not the case. Here, we used this approach to investigate whether the bias in the estimated genetic correlation indeed occurs because the marker-based relationships do not accurately predict the relationships at the causal loci, i.e., $E\left( {{\mathbf{G}}_{causal\:loci} |{\mathbf{G}}_{markers} } \right) \ne {\mathbf{G}}_{markers}$, and not because of differences in LD between populations. We calculated $b$ separately for within- and between-population relationships, using 11 bins based on pedigree relationships within populations (< 0.05, 0.05–0.10, 0.10–0.15, 0.15–0.20, 0.20–0.25, 0.25–0.30, 0.30–0.35, 0.35–0.40, 0.40–0.50, > 0.50, self-relationships) and 3 bins based on genomic relationships between populations (< − 0.10, − 0.10–0.10, > 0.10), and used those $b$ values to rescale the relationships. As shown in Fig. 8, this rescaling removed almost completely the bias in the estimated genetic correlations based on HDP and MDP markers, but overestimated the genetic correlation when using rescaled relationships based on LDP markers. This might result from the much larger sampling error for LDP markers compared to HDP and MDP markers, which could result in underestimated $b$ values. Thus, there appears to be a lower boundary for the number of markers necessary to calculate between-population genomic relationships that can be corrected using regression. Altogether, our results confirm that for an unbiased estimate of the genetic correlation between populations, the regression coefficient of true relationships on marker-based relationships should be equal to 1.

### Consistency of LD between populations

When calculating the marker-based relationships, the current generation within each population was used as the base population, since we used current population-specific allele frequencies. This means that between-population relationships were zero on average. When the LD is at least partly consistent between the populations, due to the existence of a recent or distant common ancestor, between-population relationships will show variation around zero [50]. This variation is essential to estimate the genetic correlation between populations, and genetic correlation estimates are more precise when it is larger [51].

We expected that a lower consistency of LD between populations would reduce the estimated genetic correlation between populations, because it reduces the correlation between (apparent) marker effects. Surprisingly, our results showed that estimated genetic correlations were similar with HDP and MDP markers, and only slightly lower with LDP markers. This can be explained by the potential of marker-based relationships to accurately predict the relationships at the causal loci, which is essential to estimate without bias the genetic (co)variances and the genetic correlation between populations. A lower consistency of LD between populations results in a smaller variation in between-population relationships [38, 50], both at causal loci and at markers. Therefore, the regression coefficient of the relationships at the causal loci on the relationships at the markers may not be greatly affected (Figs. 6, 7; HDP and MDP markers). Hence, the consistency of LD between the populations seems to have little impact on the estimated genetic correlation between populations.

The consistency of LD between populations does affect the correlation between the relationships at the causal loci and at the markers (Figs. 6, 7), i.e., the accuracy of the marker-based relationships. For an unbiased estimate of the genetic correlation between populations, the regression of true relationships on marker-relationships should be equal to 1 and marker-based relationships do not necessarily have to be accurate. This contrasts with the estimation of genetic values, as in genomic prediction, for which relationships must be accurate and must show variation [38]. Thus, an unbiased estimate of the genetic correlation between populations does not guarantee that accurate genomic prediction across populations can be performed.

In our study, the analysis with the HDP markers represented a situation with the highest consistency of LD between populations, and the analysis with LDP markers represented a situation with the lowest consistency of LD between populations. Therefore, the effects of marker density and consistency of LD between populations were confounded. The combined impact of marker density and consistency of LD appears to be limited, because the bias in the HDP and MDP scenarios was similar and only a little stronger in the LDP scenario. The impact of marker density can be reduced by regressing the genomic relationships towards the pedigree relationships. If the causal loci are known and the regression coefficients are calculated using relationships at causal loci, we showed that this regression completely removed the bias in estimated genetic correlations based on HDP and MDP markers. This suggests that the slight bias in the HDP and MDP scenario was due to marker density, and that differences in LD between the populations had almost no effect.

### Simulated population versus livestock populations

In order to investigate whether the simulated genome represented livestock genomes, we compared the distribution of allele frequencies and LD pattern with real genomes, as suggested by Daetwyler et al. [52]. The simulated genome showed a comparable pattern of allele frequencies between markers and causal loci, and a comparable extent and consistency of LD between populations as shown in chicken and pig populations [20, 28, 30, 42–44]. Thus, our results can be translated directly to livestock populations if the same marker density is used. This simulated LD was much higher than that generally observed in human populations [53, 54]. Since marker density, and thereby the average LD between causal loci and nearest marker, had almost no effect on the estimated genetic correlation, it is expected that the simulated LD pattern will not affect the results.

We simulated causal loci that were spread randomly across the genome, which is not always the case in real populations. When causal loci are enriched in regions with either high or low LD, (co)variance estimates can be over- or underestimated [46, 55]. However, we expect that the impact of the heterogeneity of LD will be smaller on the estimated genetic correlation than on the heritability, since differences in LD across the genome affect both the genetic variance and covariance estimates. This mechanism may also explain why estimates of the genetic correlation between traits within a population are less affected by incomplete LD between causal loci and markers than genetic variance estimates [56].

As explained above, the genetic correlation is equal to the correlation between allele substitution effects when allele substitution effects are independent of allele frequency. Differences in allele substitution effects between populations result from non-additive genetic effects and from differences in allele frequencies, and/or from genotype by environment interactions [1–3]. To date, the magnitude of these additive, dominance and epistatic effects is not well known. Therefore, we chose to simulate directly the different allele substitution effects from a bivariate normal distribution, instead of simulating the underlying non-additive effects.

Contrary to our simulations, selection generally occurs in livestock populations. Selection creates negative correlations between causal loci, known as the Bulmer effect [57]. However, the impact of the Bulmer effect on the correlation between loci is very small because the number of causal loci ($n_{causal\:loci}$) is large for most breeding goal traits (the average correlation is at maximum $\frac{ - 1}{{n_{causal\:loci} - 1}}$). Moreover, in general, selection acts on multiple traits, which further reduces the correlation between the causal loci affecting a trait. Therefore, the Bulmer effect will have only a small effect on the correlation between loci in one population. Since selection is within population, the Bulmer effect does not cause covariances between loci in different populations. For these reasons, we do not expect that selection and the Bulmer effect would have a large impact of on the results of our study.

Furthermore, the Bulmer effect is a transient phenomenon that depends on the type and intensity of selection. Hence, the additive genetic variance as affected by the Bulmer effect does not represent a fundamental biological property of a population, but it can result from selection decisions that may fluctuate over time. The biologically relevant quantity is the genic (co)variance, which is always twice the Mendelian sampling (co)variance. The relevance of the genic (co)variance follows from the decomposition of the additive genetic value of an individual into the Mendelian sampling deviations of its ancestors [58], ${\mathbf{A}} = {\mathbf{c}}^{\prime } {\mathbf{m}}$, where ${\mathbf{c}}$ is a vector of contributions of ancestors to the individual (including the individual itself, for which $c_{i} = 1$), and ${\mathbf{m}}$ is a vector of Mendelian sampling deviations of those ancestors. Hence, the variance of the additive genetic values equals $V_{A} = {\mathbf{c}}^{\prime } {\text{var}}\left( {\mathbf{m}} \right){\mathbf{c}}$. In the absence of selection, ${\text{var}}\left( {\mathbf{m}} \right)$ is diagonal and $V_{A} = \mathop \sum \nolimits_{i} c_{i}^{2} \sigma_{m}^{2} = \left( {1^{2} + 2 \times \frac{1}{2}^{2} + 4 \times \frac{1}{4}^{2} + \cdots } \right)\sigma_{m}^{2} = 2\sigma_{m}^{2}$, where $\sigma_{m}^{2}$ is the (full) Mendelian sampling variance. Selection causes ${\text{var}}\left( {m_{i} } \right)$ to deviate from $\sigma_{m}^{2}$ and creates covariances between Mendelian sampling deviations of different ancestors. These deviations are transient, and are eroded by recombination when selection ceases, so that $V_{A}$ returns to $2\sigma_{m}^{2}$ [57]. This illustrates that the genic (co)variance is the biologically relevant quantity. We have investigated the estimation of the genic correlation, by focusing on a population in the absence of selection, so that genic and genetic correlations are equal.

### Implications

Generally, marker panels are designed such that the markers have intermediate allele frequencies across multiple populations [27–29]. Hence, markers tend to have a higher average minor allele frequency than causal loci [32, 33]. Moreover, the difference in allele frequencies of causal loci between populations is probably not accurately represented by markers. These factors likely result in underestimated genetic correlations between populations using real data, but the impact of each of the factors requires further research.

## Conclusions

For an unbiased estimate of the genetic correlation between populations based on marker information, it is important that marker-based relationships accurately predict the relationships at causal loci, i.e., $E\left( {{\mathbf{G}}_{causal\:loci} |{\mathbf{G}}_{markers} } \right) = {\mathbf{G}}_{markers}$. This is obtained when the difference in allele frequencies between the two populations is similar for the markers and the causal loci, and the number of markers is sufficiently large to constrain the sampling error on between-population relationships to an acceptable level. Our results show that differences in LD phase between causal loci and markers across populations have little effect on the estimated genetic correlation (Additional file 2).

## Additional files

**Additional file 1.** Allele frequency distribution of markers and causal loci. **Figure S1:** Allele frequency distribution of HDP markers for the two populations. **Figure S2:** Allele frequency distribution of causal loci for the two populations.

**Additional file 2.** Programs and seeds to simulate data. This file contains the input file used for QMSim, the Fortran-programs to select markers and causal loci for the different scenarios, the Fortran-program to simulate phenotypes and the seeds for the different programs in each of the replicates.
