# Supplementary material for: Required properties for markers used to calculate unbiased estimates of the genetic correlation between populations
Source: Genet Sel Evol. 2018 Dec 14;50:65. doi: 10.1186/s12711-018-0434-6 (PMC6295113; doi:10.1186/s12711-018-0434-6)
Supplement: Supplementary file 1 — Additional file 1. Allele frequency distribution of markers and causal loci. Figure S1: Allele frequency distribution of HDP markers for the two populations. Figure S2: Allele frequency distribution of causal loci for the two populations. [file 12711_2018_434_MOESM1_ESM.docx]

**Additional File S1**

**
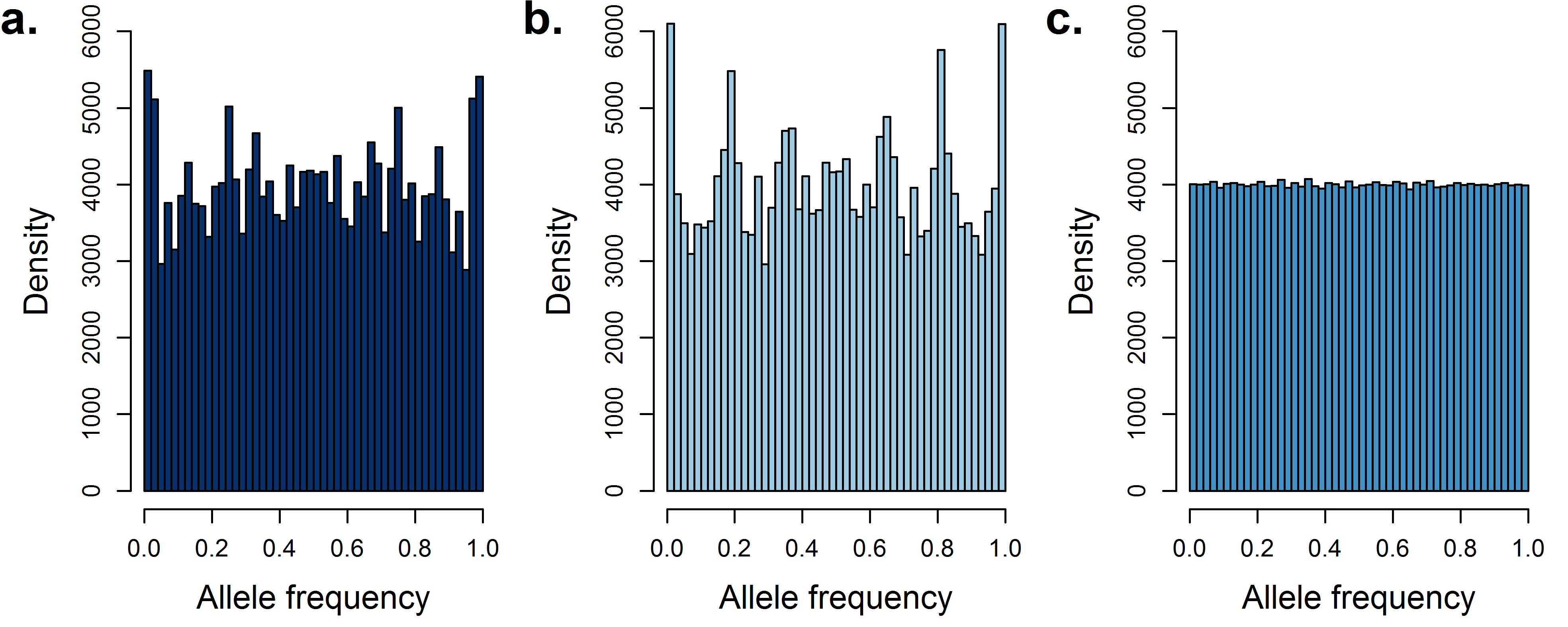
Figure S1.1 - Allele frequency distribution of HDP markers for both populations.**

Distribution of allele frequencies of selected markers of the HDP scenario for (**a.**) population A, (**b.**) population B, and (**c.**) the average of both populations for one of the replicates.


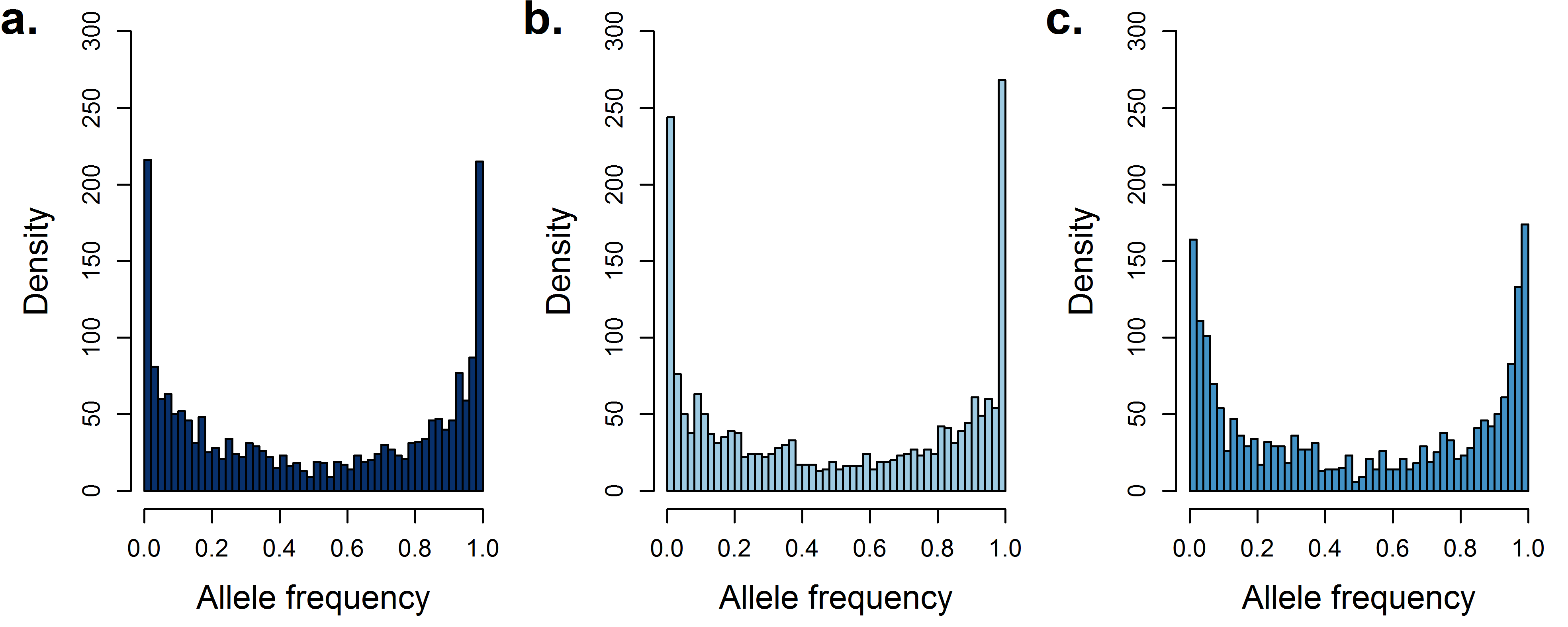


**Figure S1.2 - Allele frequency distribution of causal loci for both populations.**

Distribution of allele frequencies of selected causal loci for (**a.**) population A, (**b.**) population B, and (**c.**) the average of both populations for one of the replicates.
